# *Aedes aegypti* Piwi4 Structural Features Are Necessary for RNA Binding and Nuclear Localization

**DOI:** 10.3390/ijms222312733

**Published:** 2021-11-25

**Authors:** Adeline E. Williams, Gaurav Shrivastava, Apostolos G. Gittis, Sundar Ganesan, Ines Martin-Martin, Paola Carolina Valenzuela Leon, Ken E. Olson, Eric Calvo

**Affiliations:** 1 Laboratory of Malaria and Vector Research, National Institute of Allergy and Infectious Diseases, National Institutes of Health, Rockville, MD 20852, USA; adeline.williams@colostate.edu (A.E.W.); gaurav.shrivastava@nih.gov (G.S.); gittisa@niaid.nih.gov (A.G.G.); sundar.ganesan@nih.gov (S.G.); ines.martin-martin@nih.gov (I.M.-M.); paolacarolina.valenzuelaleon@nih.gov (P.C.V.L.); 2 Center for Vector-Borne Infectious Diseases, Department of Microbiology, Immunology, and Pathology, Colorado State University, Fort Collins, CO 80523, USA

**Keywords:** Piwi4, *Aedes aegypti*, piRNA, RNA interference, RNAi, Piwi, NLS, arbovirus, mosquito

## Abstract

The PIWI-interacting RNA (piRNA) pathway provides an RNA interference (RNAi) mechanism known from *Drosophila* studies to maintain the integrity of the germline genome by silencing transposable elements (TE). *Aedes aegypti* mosquitoes, which are the key vectors of several arthropod-borne viruses, exhibit an expanded repertoire of Piwi proteins involved in the piRNA pathway, suggesting functional divergence. Here, we investigate RNA-binding dynamics and subcellular localization of *A. aegypti* Piwi4 (AePiwi4), a Piwi protein involved in antiviral immunity and embryonic development, to better understand its function. We found that AePiwi4 PAZ (Piwi/Argonaute/Zwille), the domain that binds the 3′ ends of piRNAs, bound to mature (3′ 2′ O-methylated) and unmethylated RNAs with similar micromolar affinities (K_D_ = 1.7 ± 0.8 μM and K_D_ of 5.0 ± 2.2 μM, respectively; *p* = 0.05) in a sequence independent manner. Through site-directed mutagenesis studies, we identified highly conserved residues involved in RNA binding and found that subtle changes in the amino acids flanking the binding pocket across PAZ proteins have significant impacts on binding behaviors, likely by impacting the protein secondary structure. We also analyzed AePiwi4 subcellular localization in mosquito tissues. We found that the protein is both cytoplasmic and nuclear, and we identified an AePiwi4 nuclear localization signal (NLS) in the N-terminal region of the protein. Taken together, these studies provide insights on the dynamic role of AePiwi4 in RNAi and pave the way for future studies aimed at understanding Piwi interactions with diverse RNA populations.

## 1. Introduction

The P-element-induced wimpy testis (PIWI)-interacting RNA (piRNA) pathway is an RNA interference (RNAi) mechanism that is traditionally known to silence transposable elements (TEs) that can integrate into the germline genome and threaten its integrity [1,2,3,4]. piRNAs, 23–30 nucleotide (nt) small RNAs (sRNAs), bind Piwi proteins, a subfamily of the Argonautes. piRNA-bound Piwis assemble into piRNA-induced silencing complexes (piRISCs), where effector Piwis are targeted to complementary RNA substrates [5,6,7]. *Drosophila* express three Piwis (Piwi, Aubergine (Aub), and Argonaute-3 (Ago3)), where Aub and Ago3 are expressed exclusively in the germline, while Piwi is also expressed in neighboring somatic cells [8,9,10]. Aub and Ago3 silence their targets post-transcriptionally in the cytoplasm, while Piwi translocates into the nucleus, forms a nuclear effector complex, and silences its targets co-transcriptionally [11,12,13]. Depletion of the piRNA pathway in *Drosophila* leads to TE insertion accumulation, DNA damage, defects in embryonic development, and female sterility [14,15,16,17,18,19].

The functions of the piRNA pathway are more diverse than initially thought [3,20,21,22,23]. For example, Piwis display differential expression patterns in the germline or soma as well as in the cytoplasm or the nucleus across organisms [21], suggesting that their roles and functions are broad. A good example is in arthropods, where both somatic and germline piRNAs are common [24]. Culicine mosquitoes, specifically, have undergone an expansion of their Piwi protein repertoire, suggesting functional divergence [25]. For example, *Aedes* express seven Piwis, Piwi2-7, and Ago3, where Piwi4-6 and Ago3 are abundantly expressed in both the soma and germline [25,26]. Many studies have shown that the piRNA pathway in *Aedes* is multi-functional and important for, in addition to transposon silencing, antiviral immunity [25,27,28,29,30,31,32,33,34,35,36,37,38], embryonic development [39,40], and gene regulation [41,42].

A particularly interesting *A. aegypti* Piwi of unknown function is Piwi4 (henceforth termed “AePiwi4”). Although unnecessary for small RNA production, AePiwi4 associated with Semliki Forest virus-specific small-interfering RNAs (vsiRNAs) and virus-specific piRNAs (vpiRNAs) in infected cells, as well as with several protein players involved in both the siRNA (Ago2 and Dcr2) and piRNA (Ago3, Piwi5, Piwi6, Yb, vreteno, Tejas, and minotaur) pathways [26,43]. Furthermore, silencing *AePiwi4* depleted 3′ 2′ O-methylated (mature) Sindbis virus (SINV)-specific vsiRNAs and vpiRNAs and increased SINV, dengue (DENV2), and chikungunya (CHIKV) virus replication in infected cells [32]. This phenotype was recapitulated in DENV2-infected *A. aegypti* mosquitoes, where silencing *AePiwi4* increased infectious virus titers 5–10 days post-infection (dpi) [32]. AePiwi4 also associated with highly conserved satellite repeat-derived piRNAs (tapiR1 and tapiR2) that were 3′ 2′ O-methylated [39]. Knocking down *AePiwi4* reduced tapiR1 and tapiR2 transcripts, and depleting tapiR1 in embryos arrested their development and prevented the degradation of maternally deposited transcripts [39]. Taken together, the role(s) of AePiwi4 appear to be diverse and span across several different RNAi pathways.

AePiwi4 has been consistently associated with long (28–30 nt), mature 3′ 2′ O-methylated (henceforth termed “3′m”) piRNAs, and it was found in both the cytoplasmic and nuclear fractions in an embryonic *Aedes aegypti* cell line (Aag2) [26,32,39]. We therefore set out to characterize AePiwi4 structural motifs involved in piRNA binding and nuclear localization to gain further insights on AePiwi4 function. In human Piwis (Hiwi1, Hiwi2, and Hili), the PAZ (Piwi/Argonaute/Zwille) domain preferentially binds 3′m piRNAs because of a hydrophobic-binding pocket that is flexible enough to accommodate the methyl group [44]. This contrasts with the human Argonaute-1 (Ago1) PAZ domain where its more restrictive RNA-binding pocket exhibits preferential binding to 3′ 2′-OH (henceforth termed “3′nm”) groups present on microRNAs [44]. We hypothesized that a flexible AePiwi4 PAZ domain would also determine AePiwi4 preferential binding to mature, long piRNA populations.

We first compared PAZ sequences across previously crystalized Piwis to determine AePiwi4 PAZ structural features and binding pockets involved in 3′ end piRNA recognition. We then characterized recombinant AePiwi4 PAZ-binding dynamics with the 3′ ends of mature and un-methylated piRNAs by surface plasmon resonance (SPR). We found that mutating putative RNA-binding residues depleted or significantly impacted binding to both 3′m and 3′nm sRNAs, while a T41R change, present in *A. aegypti* Ago3, significantly improved binding. Finally, we characterized a functional nuclear localization signal (NLS) in the N-terminal region of the AePiwi4 protein. We found that subtle structural differences across Piwi proteins may have important impacts on preferential RNA-binding behaviors and subcellular localization.

## 2. Results

### 2.1. Biophysical Properties of AePiwi4 by Structural Modeling and Alignment

**Figure 1:**
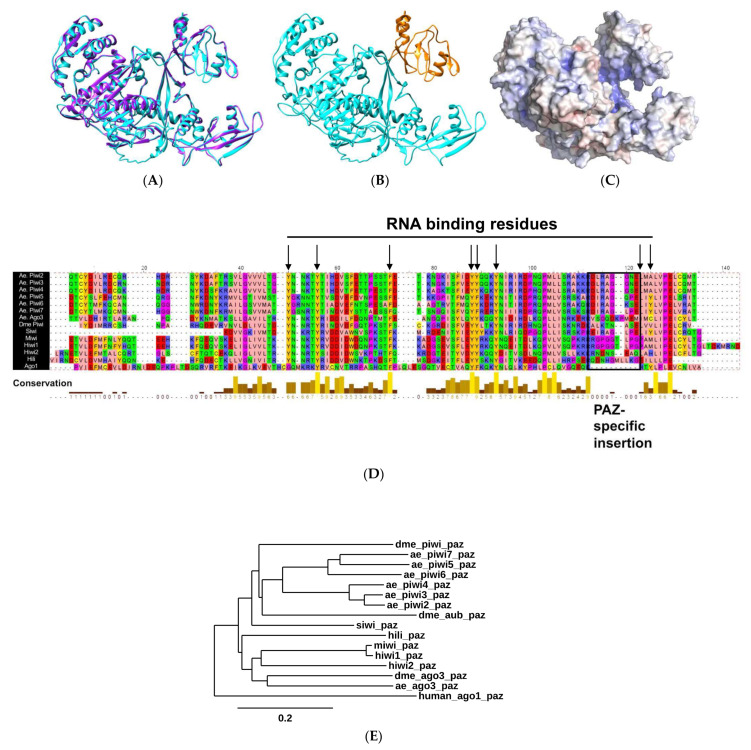
Predicted *A. aegypti* Piwi4 RNA-binding properties. (**A**) Predicted *A. aegypti* Piwi4 model (blue) superimposed with the crystalized *Drosophila* Piwi structure (purple); (**B**) predicted *A. aegypti* Piwi4 structure (blue) with PAZ domain highlighted in orange; (**C**) electrostatic density of *A. aegypti* Piwi4 where red = negatively charged and blue = positively charged. Structure is rotated on the right to reveal inner positively charged pocket; (**D**) alignment of Piwi PAZ domains, including all *A. aegypti* Piwis (Ae. Piwi2-7 and Ae. Ago3) and crystalized or characterized Piwi PAZ (*Drosophila* (Dme) Piwi, silkworm Piwi (Siwi), mouse Piwi (Miwi), and human Piwis Hiwi1, Hiwi2, and Hili). The human Argonaute protein Ago1 was also included as an outgroup. Black arrows indicate known RNA-binding residues by crystal structures, which in this alignment include amino acid numbers 50, 56, 71, 88, 89, 93, 123, and 125. Black box indicates the Piwi PAZ-specific insertion site. Colors (Zappo color scheme) indicate biochemical properties where peach = aliphatic/hydrophobic, aromatic = orange, blue = positively charged, red = negatively charged, green = hydrophilic, pink = conformationally special, and yellow = cysteine; (**E**) phylogenetic tree of all Piwi PAZ included in the alignment shown in 1D, with the addition of *Drosophila* Aubergine and Ago3 PAZs. Scale bar indicates number of substitutions per site.

Using I-TASSER and Chimera, we first modeled AePiwi4 against the recently crystalized *Drosophila melanogaster* Piwi protein [45] (PDB: 6KR6) (Figure 1A). The quality of the predicted model was assessed by its C-score = −1.50. C-scores fall between −5 and 2, and more than 90% of the quality predictions are correct for models that have C-scores of −1.5 or higher [46]. Furthermore, the average template modeling (TM) score (0.53 ± 0.15) was >0.5, which indicates a model of correct topology [46]. We then superimposed the AePiwi4 model against crystalized human (Hiwi1; PDB: 3O7V) [44] and mouse (Miwi; PDB: 2XFM) [47] PAZ to determine the amino acid residues of AePiwi4 PAZ to be M270 –T380 (Figure 1B). A summary of predicted biophysical properties of AePiwi4 PAZ is provided in Supplemental Table S1. An electrostatic density map of Piwi4 (Figure 1C) revealed an inner pocket that was highly positively charged, analogous to the *Drosophila* Piwi linker regions that bind RNA nucleotides. AePiwi4 PAZ also displayed long stretches of flexibility with neighboring hydrophobic regions (Supplemental Figure S1). The AePiwi4 PAZ model suggests that the protein contains hydrophobic regions buried within a flexible protein structure, allowing AePiwi4 to bind long 3′m piRNAs.

To determine putative AePiwi4 PAZ amino acids involved in RNA binding, we aligned Piwi PAZ sequences derived from proteins whose structures had been crystalized, including *Drosophila* Piwi [45] (PDB: 6KR6), silkworm Piwi (Siwi [48]) (PDB: 5GUH), mouse Piwi (Miwi [47]) (PDB: 2XFM), and human Hili (PDB: 3O7X) and Hiwi1 (PDB: 3O6E) [44] (Figure 1D). We also included human Hiwi2 PAZ, as its binding properties to the 3′ ends of piRNAs were characterized by isothermal calorimetry in Tian et al., 2011 [44]. As a comparison, we included the outgroup Argonaute protein human Ago1 (PDB: 4KXT), whose more restricted PAZ domain dictates its RNA-binding preference for 3′nm microRNAs [44,49]. We then compared known RNA-binding residues from the crystal structures with the residues of *A. aegypti* PAZ domains (black arrows, Figure 1D; black arrows, Supplemental Figure S1). We found that the residues involved in RNA binding tended to be highly conserved across the different organisms, and many were tyrosine and phenylalanine aromatic residues, whose hydroxyl groups form hydrogen bonds with phosphate oxygens of RNA nucleotides [44]. We also noted that all Piwi PAZ domains analyzed herein displayed the Piwi PAZ specific insertion element (black box, Figure 1D) that provides the flexibility necessary for accommodating 3′m piRNA ends [44]. This insertion site lies between two beta barrels, which, when absent (as in Ago1), results in a sharp turn between the barrels and a narrower binding pocket [44]. Although the amino acids within the Piwi PAZ-specific insertion site are not conserved across organisms, or even across subfamily Piwi proteins in the same organism [44], we observed that the first five amino acids of the Piwi PAZ-specific insertion sites were highly conserved across the *A. aegypti* Piwis. The only exception to this observation was *A. aegypti* Ago3, whose residues shared no similarity to its AePiwi PAZ counterparts. Further, Ago3 displayed two amino acids within the insertion site not seen in the other PAZ sites, perhaps suggesting a more flexible binding pocket than the other *A. aegypti* Piwis.

We also generated a phylogenetic tree to compare evolutionary relatedness between Piwi PAZ sequences from the various organisms (Figure 1E). We included human Ago1 again as an outgroup. We also added the two additional *Drosophila* Piwis, Aub, and Ago3. We found that all *A. aegypti* Piwi PAZ, except for Ago3, clustered with *Drosophila* Aub. On the other hand, *A. aegypti* Ago3 was most closely related to *Drosophila* Ago3. We also observed AePiwi4 clustered with the germline AePiwis Piwi2-3 as opposed to the somatic AePiwis Piwi5, Piwi6, and Ago3.

### 2.2. A. aegypti Piwi4 PAZ Binds 3′ 2′ O-Methylated and Non-Methylated piRNAs in a Sequence-Independent Manner

To determine whether *A. aegypti* Piwi4 PAZ bound to both 3′ 2′ O-methylated and 3′ 2′ OH piRNAs, we cloned *AePiwi4 PAZ* with a histidine 6xHis-tag into pET-17b for bacterial expression (Supplemental Figure S2A,B), purified it, and characterized RNA-binding dynamics by SPR. We confirmed AePiwi4 PAZ expressed in BL21(DE3) pLysS *E. coli* by Western blot using antibodies against its 6xHis-tag (Supplemental Figure S2C). We purified AePiwi4 PAZ from the soluble fraction by nickel chromatography followed by size exclusion chromatography. Soluble AePiwi4 PAZ was stable in 20 mM Tris-HCl pH 7.4 and 150 mM NaCl and ran at the expected size (14 kDa) by SDS/PAGE gel electrophoresis (Supplemental Figure S2D). Protein identity was confirmed by Edman degradation.

To test RNA-binding dynamics, we performed SPR with AePiwi4 PAZ and three different RNAs: a 3′m 28 nt Phasi Charoen-like virus (PCLV)-specific piRNA, a 3′nm vpiRNA of the same sequence, and a 3′nm 28 nt scrambled sequence. We chose this piRNA sequence because a recent publication noted that PCLV piRNAs are broadly distributed across culicine mosquito cell lines, perhaps due to a PCLV-specific endogenous viral element in the genome [50]. Using synthetic RNA sequences biotinylated on the 5′ end, we immobilized the RNA on a CM5 Biacore surface that had been pre-coated with neutravidin. Immobilizing RNA by the 5′ end allowed us to test binding affinities to moieties at the 3′ end. Using increasing concentrations of AePiwi4 PAZ analytes flowed over the surface of the chip with immobilized ligand, we were able to determine the dissociation constants (K_D_) from steady-state binding levels (R_eq_) against the analyte concentration (C, in molar concentration) once binding reached equilibrium. Experiments were performed in four replicates.

**Figure 2:**
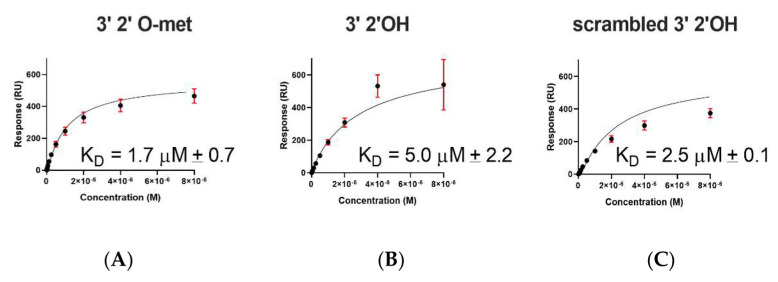
Affinity-binding equilibrium curves for AePiwi4 PAZ to piRNAs. Fitted affinity-binding equilibrium curves for AePiwi4 PAZ analyte to a 28 nt (**A**) 3′ 2′ O-methylated, (**B**) non-methylated, or (**C**) scrambled 28 nt non-methylated piRNA. Equilibrium K_D_ was calculated from steady-state binding levels R_eq_ = (CR_max_)/(K_D_ + C) + offset, where C = concentration, R_max_ = analyte-binding capacity of the surface in response units (RU), K_D_ = dissociation constant, and offset = response at zero analyte concentration. M = molar concentration. Red bars indicate mean and standard deviation for R_max_ values.

We found that AePiwi4 PAZ bound the 3′m 28 nt piRNA with a K_D_ of 1.7 ± 0.8 μM (Figure 2A), the 3′nm 28 nt piRNA with a K_D_ of 5.0 ± 2.2 μM (Figure 2B), and the scrambled 28 nt 3′nm piRNA with a K_D_ of 2.5 ± 0.1 μM (Figure 2C). AePiwi4 PAZ bound to 3′ 2′ O-methylated piRNAs with marginally greater affinity than it did to 3′ 2′ unmethylated piRNAs (*p* = 0.05), and there was no significant difference in binding affinities for known or scrambled RNA sequences (*p* = 0.25).

### 2.3. A. aegypti Piwi4 PAZ Mutants Reveal the Amino Acids Necessary for piRNA Binding

**Figure 3:**
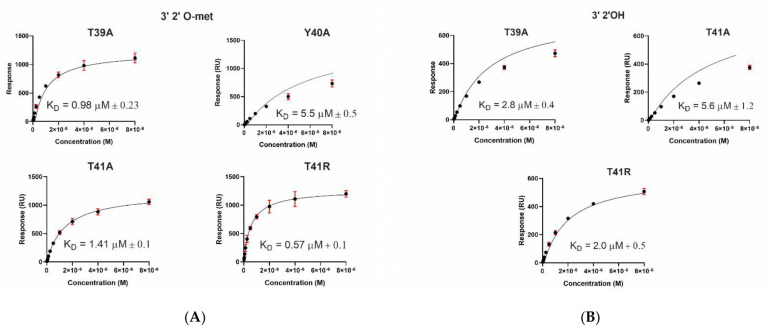
Affinity-binding equilibrium curves for AePiwi4 PAZ mutants to piRNAs. Fitted affinity-binding equilibrium curves for AePiwi4 PAZ mutant analytes to a 28 nt (**A**) 3′ 2′ O-methylated or (**B**) non-methylated piRNA. Equilibrium K_D_ was calculated from steady-state binding levels R_eq_ = (CR_max_)/(K_D_ + C) + offset, where C = concentration, R_max_ = analyte-binding capacity of the surface in response units (RU), K_D_ = dissociation constant, and offset = response at zero analyte concentration. M = molar concentration. Red bars indicate mean and standard deviation for R_max_ values.

For further insights into how the AePiwi4 PAZ structure dictates its RNA-binding preferences, we generated AePiwi4 PAZ mutants that displayed amino acid changes within predicted RNA-binding pockets. We focused our efforts around two highly conserved residues shown to form hydrogen bonds with RNA—Y40 and F55—as well as two residues that flank Y40 and appeared to be moderately conserved across the *A. aegypti* Piwi PAZ—T39 and T41. Through site-directed mutagenesis, we generated five mutants that we then expressed in bacteria and purified: T39A, Y40A, T41A, F55A, and T41R (Supplemental Figures S3 and S4). Y40A and F55A displayed alanine substitutions for the highly conserved tyrosine or phenylalanine amino acids, respectively, while T39A and T41A displayed alanine substitutions for the threonines that flank Y40. The T41R mutation reflected the arginine present in only one *A. aegypti* Piwi, Ago3, but also in most other organisms’ Piwi PAZ domains analyzed herein (Figure 1D). Inserts were confirmed by Sanger sequencing, and mutations in protein sequence were confirmed by mass spectrometry. Binding behaviors were assessed by SPR, as described previously (Figure 3).

Dissociation constants for all mutant proteins binding both the 3′m and 3′nm 28 nt piRNA are displayed in Figure 3 and summarized in Table 1. We found that F55 was essential for both 3′m and 3′nm binding because when mutated, no binding occurred for either ligand. Y40A also depleted 3′nm binding and significantly inhibited 3′m piRNA binding (K_D_ = 5.5 ± 0.5 μM; *p* = 0.04). We found that disrupting the amino acids flanking Y40 with alanine mutations had no significant impact on binding 3′m piRNAs as compared to wild-type PAZ binding to this RNA (T39A: *p* = 0.2; T41A: *p* = 0.3). However, we did observe a significantly increased affinity of T39A for the 3′nm piRNA (K_D_ = 2.8 ± 0.4 μM; *p* = 0.02), suggesting that this residue does have an impact on 3′nm binding. Furthermore, we observed that mutating T41 to match the amino acid present in *A. aegypti* Ago3 PAZ tended to improve 3′m binding (K_D_ = 0.57 ± 0.1 μM) and significantly improved 3′nm binding (K_D_ = 2.0 ± 0.5 μM; *p* = 0.02).

**Figure 4:**
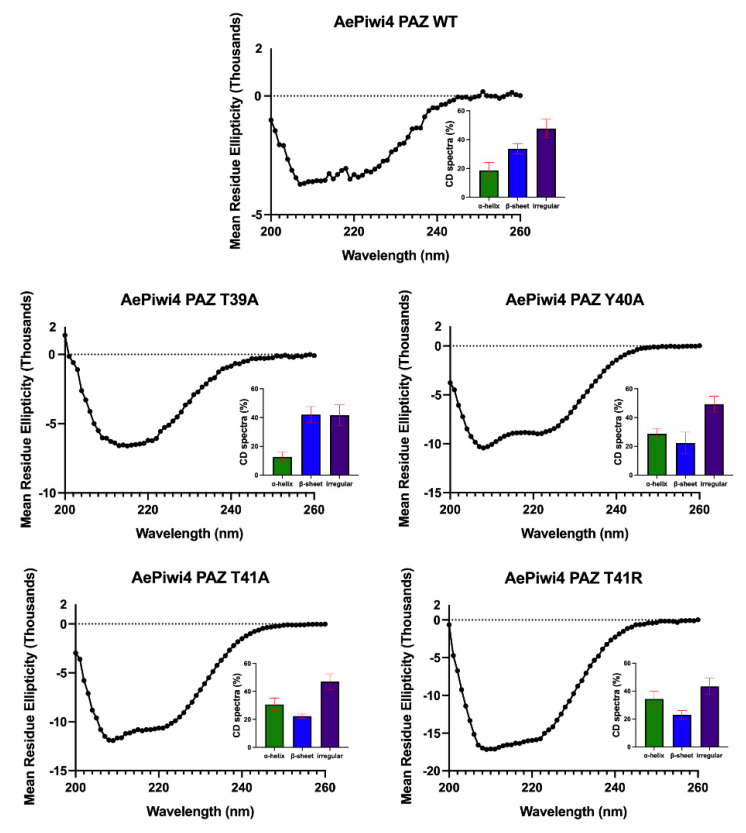
Circular dichroism (CD) spectra analyses of AePiwi4 PAZ mutants. CD spectra curves, by mean residue ellipticity for AePiwi4 PAZ WT and mutant proteins, recorded over 200–260 nm. Insets show the calculated percentages of secondary structures determined by CD analysis using CAPITO. Red bars indicate mean and standard deviation of three similarity hits based on lowest area differences under the curve.

In Hiwi1 PAZ, preferential binding of 3′m RNA over 3′nm RNA is mostly dictated by backbone confirmation of the protein rather than the amino acid composition of the binding pocket [44]. To investigate whether the mutations that impacted the RNA binding impacted the AePiwi4 PAZ secondary structure, we performed circular dichroism (CD) spectroscopy analysis with the T39A, Y40A, T41A, and T41R mutants and compared the CD curves to that of the WT AePiwi4 PAZ (Figure 4). We analyzed the data using CAPITO [51]. WT AePiwi4 PAZ displayed a CD curve most similar to proteins that had a mostly irregular structure but also that had between 30% and 49% beta strands and 6–16% alpha helices. All mutants maintained a mostly irregular secondary structure; however, they displayed different CD curves and percentages of alpha helices and beta-sheets compared to the WT protein (Figure 4 insets). T41R was most similar to proteins that were made up of between 16% and 26% beta strands and 28–46% alpha helices. Y40A also displayed a spectrum that aligned more with proteins that had a greater abundance of alpha helices—26–40% alpha helices but only 14–22% beta-strands. The T39A CD curve clustered with proteins that were made up of 9–25% alpha helices and 30–41% beta-strands, while the T41A curve clustered with proteins that were made up of 31–50% alpha helices and 4–21% beta-strands. Taken together, these data indicate that single amino acid changes in the AePiwi4 PAZ backbone can alter secondary structure, which likely impacted RNA-binding behaviors.

### 2.4. AePiwi4 Co-Localizes in the Cytoplasm and Nucleus in A. aegypti Tissues

For further insights into the function of *A. aegypti* Piwi4, we characterized the sub-cellular localization of the native protein in *A. aegypti* mosquitoes. While this manuscript was under preparation, Joosten and colleagues (2021) reported that Piwi4, Piwi5, and Piwi6 were in both the nucleus and the cytoplasm in an *A. aegypti* embryonic cell line infected or uninfected with Sindbis virus [26]. To determine the sub-cellular localization of native AePiwi4 in both somatic and germline tissues in the mosquito, we generated polyclonal antibodies against AePiwi4 by immunizing mice with the AePiwi4 PAZ recombinant protein. We confirmed that these antibodies recognized both recombinant AePiwi4 PAZ and full-length proteins by Western blot (Supplemental Figure S5). To confirm that the antibodies recognized AePiwi4 from mosquito tissues, we prepared whole mosquito lysates from three *A. aegypti* females 48 h post-bloodmeal (time of peak AePiwi4 expression [32]) for Western blot. Anti-AePiwi4 mouse serum reacted to whole mosquito lysate at the expected size of AePiwi4 (100 kDa) (Supplemental Figure S5). Mass spectrometry analyses further confirmed that AePiwi4-specific peptides were present at the same location on a corresponding SDS/PAGE gel slice (Supplemental Dataset 1).

**Figure 5:**
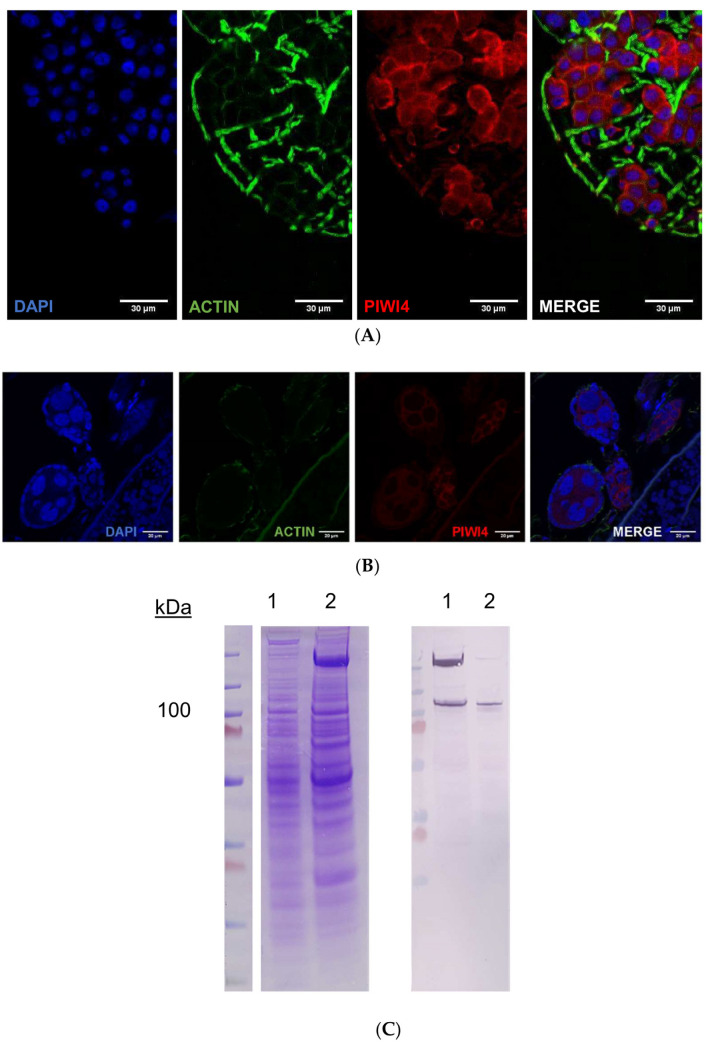
AePiwi4 expression in mosquito tissues. IFA of *A. aegypti* midgut (**A**) or ovaries with unfertilized embryos (**B**) stained with anti-AePiwi4 (red), phalloidin (green), or DAPI (blue). Scale bars are 30 μM and 20 μM for midguts and embryos, respectively; (**C**) Coomassie-stained SDS/PAGE gel (left) and corresponding Western blot of cytoplasmic (lane 1) or nuclear (lane 2) fractions of *A. aegypti* mosquito ovary tissue.

To determine the sub-cellular localization of AePiwi4, we next performed immunofluorescence assays and Western blots using both somatic and germline-derived mosquito tissues. AePiwi4 tended to stain cytoplasmically in both midguts (Figure 5A; Supplemental Figure S6A) and unfertilized embryos from ovary tissues (Figure 5B, Supplemental Figure S6B). However, when we fractionated ovaries from *A. aegypti* mosquitoes 48 h post-bloodmeal into cytoplasmic and nuclear fractions for Western blot, we found that AePiwi4 was present in both fractions (Figure 5C). Antibodies targeting H3 histone were used as a marker for the nuclear fraction (Supplemental Figure S7). These results suggested AePiwi4 may be trafficked in and out of the nucleus in mosquito tissues.

### 2.5. A. aegypti Piwi4 Expresses a Nuclear Localization Signal in the N-Terminal Region of the Protein

To further explore AePiwi4 nuclear localization, we identified a putative NLS in the N-terminal region of the protein (Supplemental Figure S8A). In *Drosophila melanogaster* Piwi, the NLS is expressed in the intrinsically disordered domain in a similar region of the N-terminal. We therefore generated a phylogenetic tree of the intrinsically disordered regions of the *A. aegypti* and *D. melanogaster* Piwi proteins to see if Piwis with known or putative NLS signals would cluster together (Supplemental Figure S8B). We found that the intrinsically disordered domains of *A. aegypti* Ago3 and *D. melanogaster* Ago3 clustered together, that *Drosophila* Aub and *A. aegypti* Piwi7 clustered together, and that *A. aegypti* Piwis2-6 clustered with *Drosophila* Piwi, the only *Drosophila* Piwi with an NLS. These results suggested that *A. aegypti* Piwi2-6 may also harbor nuclear localization signals in their intrinsically disordered domains in the N-terminal regions.

**Figure 6:**
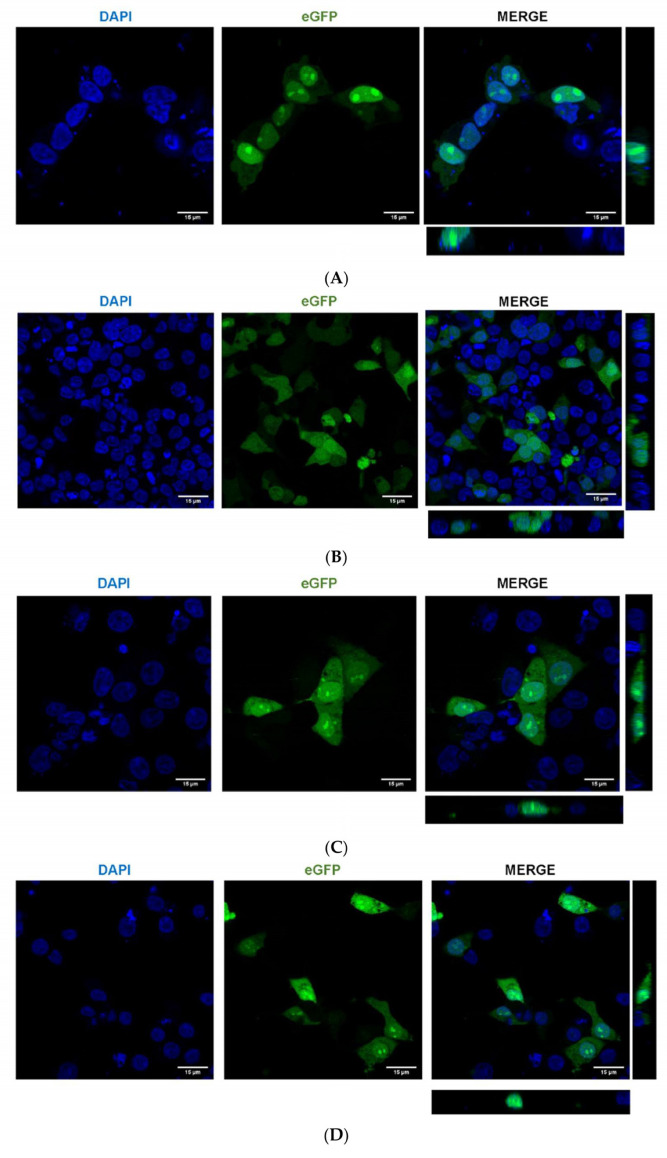

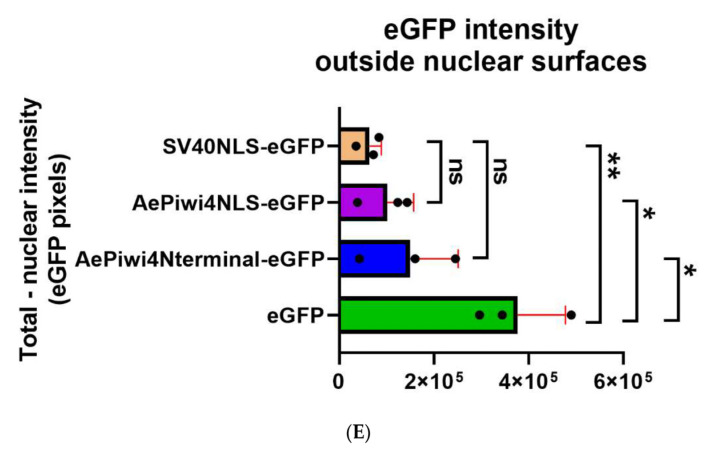
AePiwi4 harbors an NLS in the N-terminal region of the protein. Representative slices from image stacks of HEK293 cells transfected with (**A**) SV40NLS-eGFP, (**B**) eGFP, (**C**) AePiwi4NLS-eGFP, or (**D**) AePiwi4Nterminal-eGFP. Cells were stained with DAPI (blue) 24 h post-transfection. DAPI (left), eGFP (middle), and merged (right) channels are shown separately. Orthogonal views presented in merged channel. Scale bar = 15 μM. (**E**) Quantification of total eGFP fluorescence intensity sums subtracted from eGFP intensity sums in nuclear surfaces for each sample type, normalized by number of cells. Each black dot represents an individual picture. Red bars indicate SEM. ** = *p* ≤ 0.01, * = *p* ≤ 0.05, ns = non-significant.

To confirm that the putative AePiwi4 NLS was responsible for protein nuclear localization, we cloned the putative AePiwi4 NLS (amino acid residues 42–83, Supplemental Figure S8A), as well as the entire N-terminal region containing the NLS (amino acid residues 1–83), fused to an eGFP; we henceforth named these constructs AePiwi4NLS-eGFP and AePiwi4Nterminal-eGFP, respectively. We used the same backbone containing either a known SV40 NLS fused to the eGFP [52] as a positive control or an eGFP alone as a negative control; we henceforth named these constructs SV40NLS-eGFP and eGFP, respectively. We then transfected HEK293 cells with these constructs and visualized eGFP and DAPI colocalization 24 h post-transfection. As expected, we found that the known SV40NLS-eGFP localized in the nucleus while the eGFP alone appeared diffused throughout the cells (Figure 6A,B). Plasmids harboring either AePiwi4NLS-eGFP or AePiwi4Nterminal-eGFP migrated into the nucleus, as evidenced by eGFP expression colocalized with DAPI staining (Figure 6C,D). The nuclear staining appeared punctated, perhaps indicative of nucleolar staining. We also observed that both AePiwi4NLS-eGFP and AePiwi4Nterminal-eGFP displayed cytoplasmic eGFP expression as well, which was not observed in cells transfected with the SV40 NLS plasmid (Figure 6C,D).

To quantitively compare eGFP fluorescent intensities across sample types, we subtracted total eGFP fluorescent intensity sums from eGFP fluorescent intensity sums in nuclear surfaces, normalized by number of cells, in three independent views across slides (Figure 6E). We found that the resulting eGFP nuclear intensity sums outside of the nuclear surfaces was significantly higher for cells transfected with the eGFP construct as compared to cells transfected with SV40NLS-eGFP (*p* = 0.006), AePiwi4NLS-EGFP (*p* = 0.01), or AePiwi4Nterminal-eGFP (*p* = 0.05). There were no significant differences in eGFP fluorescent intensity sums outside of nuclear surfaces between cells transfected with AePiwi4NLS-EGFP (*p* = 0.34) or AePiwi4Nterminal-eGFP (*p* = 0.23) compared to those transfected with the SV40NLS-eGFP positive control. Taken together, these results suggested that *A. aegypti* Piwi4 expresses an NLS in the intrinsically disordered domain in the N-terminal region of the protein.

## 3. Discussion

In this study, we characterized *A. aegypti* Piwi4 structural features involved in RNA binding and nuclear localization to gain insights into the protein’s function. AePiwi4 had previously been associated with various 28–30 nt 3′ 2′ O-methylated piRNAs, so we focused our efforts on the PAZ domain that binds the 3′ ends of piRNAs. We assessed AePiwi4 PAZ RNA-binding dynamics by SPR and found that AePiwi4 PAZ bound to both mature and unmethylated piRNAs with micromolar affinities in a sequence independent manner. We identified key residues in AePiwi4 PAZ involved in RNA binding and found that they were highly conserved across organisms. We also highlighted a unique arginine amino acid flanking a tyrosine residue necessary for 3′nm RNA binding that was present in most other organisms’ Piwi PAZ but was only present in a single *A. aegypti* Piwi PAZ (Ago3). Mutating this residue in AePiwi4 PAZ to match that of Ago3 improved both 3′m and 3′nm RNA binding. Through circular dichroism, we showed that single amino acid changes in Piwi PAZ changes the secondary structure of the protein. Finally, we found that AePiwi4 was both cytoplasmic and nuclear in mosquito tissues, and that signals in the intrinsically disordered region drove nuclear localization.

We report herein Piwi-RNA-binding affinities for a Piwi protein of an arthropod vector, which complements studies performed with human and *Drosophila melanogaster* Piwi PAZ. We found that *A. aegypti* Piwi4 PAZ bound 3′ 2′ O-methylated and non-methylated piRNAs with K_D_s of 1.7 ± 0.8 μM and 5.0 ± 2.2 μM, respectively. The preference of AePiwi4 PAZ for 3′m piRNAs over 3′nm piRNAs was less pronounced than what has been reported for other Piwi PAZ (Supplemental Table S2). For example, Hiwi1, Hiwi2, and Hili bound 3′m piRNAs with K_D_s of 6.5 μM, 2 μM, or 10 μM, respectively, but they bound non-methylated piRNAs with weaker affinities—K_D_s of 16 μM, 12 μM, or 34 μM, respectively [44]. Immunoprecipitations of AePiwi4 from uninfected or infected Aag2 cells followed by sRNA sequencing of associated RNAs have revealed the protein associates with bona fide piRNAs resistant to beta-elimination, a method that selects for 3′m piRNAs and depletes 3′nm miRNAs [32,39]. Our results suggest, however, that AePiwi4 is able to bind to both 3′m and 3′nm sRNAs with only a marginally higher affinity for the former over the latter. Further investigations on AePiwi4-associated sRNAs from different cellular compartments may provide new insights on protein-RNA trafficking and the range of sRNAs with which AePiwi4 interacts. For example, the role(s) of *A. aegypti* Piwis may function with both pre-processed non-methylated RNAs and mature piRNAs across cellular compartments or with sRNA populations outside the piRNA pathway. Halbach et al. [39] compared AePiwi4-mediated silencing of a satellite repeat-derived target by way of a piRNA to that of miRNA silencing [39]. In that study, the authors found that the 3′ end of a satellite repeat-derived piRNA (tapR1) was not absolutely required for silencing, while the seed region was not sufficient for silencing, a pattern they compared to miRNA-mediated silencing [39]. In another study, Tassetto and colleagues found that silencing *AePiwi4* impacted both 3′m piRNA and siRNA production and argued that AePiwi4 links the siRNA and piRNA pathways [32]. Our AePiwi4 RNA-binding studies indicate that the PAZ domain of AePiwi4 is indeed able to interact with diverse populations of sRNAs with similar affinities, perhaps suggesting AePiwi4 has broad functions or unique roles in RNA binding that may differ from model Piwis. Future studies comparing RNA-binding dynamics across the *A. aegypti* Piwis will elucidate the roles they play in RNAi.

In this study, we studied 3′m and 3′nm RNA binding with protein partners by SPR. Other studies have characterized PAZ RNA binding by isothermal calorimetry (ITC) using small eight nucleotide RNAs, and we note that caution should be taken when comparing hard dissociation constant values across these different techniques. While ITC provides valuable information on number of binding sites and heat released from a binding reaction, we found that immobilizing the 5′ end of longer, more physiologically relevant RNAs by SPR enabled us to efficiently calculate dissociation constants for many protein-binding partners against stabilized ligands in a single experiment. This method may be useful for other studies aimed at understanding protein-RNA binding at specific motifs.

Our data suggest that small differences in Piwi PAZ amino acid composition across Piwi proteins alter protein secondary structure, which thereby impact the protein’s affinity for certain RNAs. Given that preferential binding of 3′m RNA over 3′nm RNA was mostly dictated by backbone confirmation of the protein in Hiwi1, it is likely that subtle differences in Piwi protein structure may have profound impacts on preferential RNA-binding behaviors that are important for defining the functions of different Piwis. We found that although the amino acids that directly form hydrogen bonds with RNA were highly conserved, residues that flank these sites tended to be more variable. Perhaps those residues that impact the stability of the PAZ structure, rather than those that directly bind the RNA itself, drive Piwi functional divergence.

The number of Piwi proteins has expanded in culicine mosquitoes as compared to anophelines and drosophilids [53], and understanding their evolutionary relationships with other Piwis, their shared or unique structural features, and their interactions with diverse RNA populations may provide insights into how their functions have diverged. We found that the PAZ domains of *A. aegypti* Piwis 2-7 are more evolutionarily related to that of *D. melanogaster* Aubergine than to *D. melanogaster* Piwi or Ago3 PAZ. Aubergine is a cytoplasmic, germline-specific protein that participates in ping-pong amplification by binding antisense primary piRNAs and producing secondary sense piRNAs that fuel the cycle [54], a role similar to that of *A. aegypti* Piwi5 [25]. A recent study showed that piRNA binding to Aub PAZ induces a protein confirmational change that triggers symmetric dimethylarginine (sDMA) methylation of the Aub intrinsically disordered domain in the N-terminal region [55]. The sDMA modification then serves as a binding site for Krimper, which simultaneously binds unmethylated Ago3 to bring the proteins in close proximity for RNA transfer during ping-pong amplification [55]. Indeed, Joosten and colleagues recently characterized a Krimper ortholog in *Aedes*, Atari, which bound Ago3 without sDMA modifications [26]. Perhaps a similar mode of RNA binding-dependent autoregulation and sDMA signaling also govern the *Aedes aegypti* Piwis Piwi2-6.

*Drosophila* Piwi, the only nuclear Piwi in the fly, expresses a bipartite NLS in its intrinsically disordered domain [56]. *Drosophila* Piwi nuclear localization is autoregulated by confirmational changes that occur once the protein binds piRNAs; the NLS remains buried within the protein structure until RNA binding triggers a conformational change and exposes the NLS [56]. Once the protein is imported into the nucleus and releases the piRNA, the protein is trafficked back into the cytoplasm. We found that *A. aegypti* Piwi4 also expresses signals in the intrinsically disordered region that drive proteins to the nucleus (Figure 6C,D), which, if similar to *Drosophila* Piwi, could autoregulate protein trafficking based on protein confirmational changes that dictate signal exposure. We observed that the AePiwi4 NLS did not drive complete expression of eGFP into the nucleus, as evidenced by diffused cytoplasmic fluorescence in addition to punctated nuclear staining (Figure 6C,D). It is possible that the intrinsically disordered region of *AePiwi4* contains both an NLS and nuclear export signals (NES) that drive protein trafficking in and out of the nucleus. Investigations on how AePiwi4 regulates its trafficking into different cellular compartments require future studies and could be useful in understanding its role in different RNAi-mediated processes. It is also possible that several *A. aegypti* Piwis autoregulate their subcellular localization in similar manners as AePiwi4. Our phylogenetic analyses revealed that the regions of the *AePiwi4* and *Drosophila melanogaster* Piwi proteins that harbored nuclear localization signals, the intrinsically disordered domains, also clustered with *A. aegypti* Piwi2, Piwi3, Piwi5, and Piwi6 (Supplemental Figure S8B). Different Piwis likely have sophisticated and diverse regulation mechanisms that control their expression patterns in different compartments of the cell.

Growing evidence reveals that the piRNA pathway is involved in gene regulation in somatic tissues and contributes to diverse human diseases including cancer [23,57] and neurodegenerative disorders [58]. Because somatic piRNAs and Piwi expression are common in arthropods [24], they could be valuable models for understanding the molecular mechanisms underlying the lesser understood Piwi or piRNA functions. Future studies aimed at understanding how Piwi-RNA binding impacts protein structure and function will be useful for learning more about how this pathway is involved in immunity, gene regulation, and disease in arthropod vectors as well as in other organisms.

## 4. Materials and Methods

### 4.1. A. aegypti Piwi4 Structure Model Prediction

A model of the *A. aegypti* Piwi4 structure was generated using the I-TASSER software (version 5.1 Zhang Lab, University of Michigan, Ann Arbor, MI, USA [46,59,60]) and visualized using Chimera (University of California, San Francisco, CA, USA). This software predicts secondary and tertiary structures based on the similarity of other proteins whose structures have been solved. The AePiwi4 amino acid sequence was queried against the *Drosophila* Piwi structure that was crystalized in Yamaguchi et al., 2020 [45], which allowed us to determine the predicted *A. aegypti* Piwi4 PAZ domain. AePiwi4 PAZ was then superimposed to other crystalized PAZ proteins, including Hili PAZ [44], Hiwi1 PAZ [44], Miwi PAZ [47], and Siwi PAZ [48].

### 4.2. Cloning

*Aedes aegypti Piwi4* (*AAEL007698*), including a 6xHis-tag, was synthesized by BioBasic Inc. (Markham, ON, Canada). Both *AePiwi4* full length (FL) and PAZ domain (residues 270–380) nucleotide sequences were sub-cloned into pCR-Blunt II-TOPO vector using the Zero Blunt TOPO PCR Cloning Kit (Thermo Fisher Scientific, Waltham, MA, USA) following the manufacturer’s instructions. pCR-Blunt II-TOPO vectors containing either *AePiwi4* FL or *AePiwi4* PAZ were transformed in OneShot Top10 chemically competent *E. coli* (Invitrogen, Waltham, MA, USA). Using standard restriction enzyme-mediated cloning and the pCR-Blunt II-TOPO vectors described above as PCR templates, *AePiwi4* FL or *AePiwi4* PAZ were then cloned into pET-17b vectors. pET-17b vectors containing either *AePiwi4* FL or *AePiwi4* PAZ were transformed in OneShot Top10 chemically competent *E. coli* (Invitrogen, Waltham, MA, USA). Inserts were confirmed by Sanger sequencing. Primers used in this study are displayed in Supplemental Table S3.

The putative AePiwi4 NLS, as well as the entire N-terminal region of AePiwi4 containing the putative NLS, were cloned from the *AePiwi4* FL-containing pCR-Blunt II-TOPO vector into a backbone containing an eGFP by In-Fusion cloning (TakaRa, San Jose, CA, USA) following the manufacturer’s instructions. The parent SV40NLS-eGFP backbone was a gift from Rob Parton (Addgene plasmid # 67652; http://n2t.net/addgene:67652 (accessed on 28 April 2021); RRID: Addgene_67652) [52]. Briefly, an eGFP-alone plasmid was generated by NcoI digestion and religation with the T4 DNA ligation Mighty Mix (TakaRa, San Jose, CA, USA) of the SV40NLS-eGFP plasmid. AePiwi4NLS-eGFP or AePiwi4Nterminal-eGFP were then cloned into the eGFP alone plasmid using In-Fusion primers listed in Supplemental Table S3. All constructs were transformed into Stellar Competent Cells (TakaRa, San Jose, CA, USA), and inserts were confirmed by Sanger sequencing.

### 4.3. Recombinant Protein Expression

pET-17b vectors containing either *AePiwi4* FL or PAZ were transformed into BL21(DE3) pLysS chemically competent *E. coli* cells (Thermo Fisher Scientific, Waltham, MA, USA). Transformed *E. coli* were plated onto Luria–Bertani (LB) agar plates with 100 µg/mL ampicillin and 34 µg/mL chloramphenicol that were left O/N at 37 °C. Individual colonies were picked into starter cultures of 4 mL LB broth (supplemented with 100 µg/mL ampicillin and 34 µg/mL chloramphenicol) that were left shaking at 220 RPM O/N at 37 °C. Starter cultures were then added to 150 mL LB broth (supplemented with 100 µg/mL ampicillin and 34 µg/mL chloramphenicol) that were left shaking at 220 RPM 37 °C until OD_600_ = 0.4 (~1 h). Protein expression was then induced with 0.1 mM isopropyl β- d-1-thiogalactopyranoside (IPTG) for 4 h shaking at 160 RPM at 25 °C. Bacteria was then pelleted and stored at −30 °C until protein purification.

For larger scale expression, 150 mL LB broth (supplemented with 100 µg/mL ampicillin and 34 µg/mL chloramphenicol) starter cultures that had been inoculated with glycerol scrapings of BL21(DE3) pLysS *E. coli* containing either pET-17b-*AePiwi4* FL or pET-17b-*AePiwi4* PAZ were left shaking at 220 RPM O/N at 37 °C. Starter cultures were then added to 1 L LB broth (supplemented with 100 µg/mL ampicillin and 34 µg/mL chloramphenicol) and expression was induced following the above protocol.

Expression was confirmed by SDS-PAGE separation and anti-6xHis-tag Western blot in both the soluble and inclusion body fractions for both proteins.

### 4.4. Recombinant Protein Purification

The soluble AePiwi4 PAZ protein was purified by affinity chromatography followed by size-exclusion chromatography using Nickel-charged HiTrap Chelating HP (GE Healthcare, Chicago, IL, USA) and Superdex 200 10/300 GL columns (GE Healthcare, Chicago, IL, USA), respectively. Frozen *E. coli* pellets were resuspended with Buffer A (10 mM Tris, 500 mM NaCl, 5 mM imidazole, pH 8), left on ice for 10–15 min, and pulse sonicated 4× for 30 s—2 min. The lysates were then spun at 15,000× *g* for 30 min at 4 °C. The resulting supernatants were filtered with a 0.8 µM filter (MilliporeSigma, Burlington, MA, USA) and loaded onto a pre-equilibrated Nickel-charged HiTrap Chelating HP column using a peristaltic pump. The column was then pre-washed with 3 column volumes (CV) of Buffer A, followed by a 3 CV wash with Wash Buffer 1 (10 mM Tris, 500 mM NaCl, 20 mM imidazole, pH 8) and 3 CV wash with Wash Buffer 2 (10 mM Tris, 500 mM NaCl, 100 mM imidazole, pH 8). The protein was eluted from the column using 3 CV of Elution Buffer (10 mM Tris, 500 mM NaCl, 300 mM imidazole, and pH 8) and visualized by SDS-PAGE gel electrophoresis.

Eluted protein was concentrated down to ~500 µL using an Amicon stirred cell with a cellulose membrane of 3 kDa nominal molecular weight (MilliporeSigma, Burlington, MA, USA). The resulting concentrated protein was spun down at 4000× *g* for 10 min to remove large debris and loaded onto a Superdex 200 10/300 GL column that had been equilibrated with 20 mM Tris-HCl, 150 mM NaCl, pH 7.4. Peak elutions that corresponded to the correct size of Piwi4 PAZ (14 kDa) were confirmed by SDS-PAGE gel electrophoresis. N-terminal protein sequence was also confirmed by Edman degradation.

### 4.5. SDS-PAGE

All proteins were heated to 95 °C for 5 min under reducing conditions in 1X LDS (Thermo Fisher Scientific, Waltham, MA, USA) and were separated using 4–12% Bis-Tris protein gels (Thermo Fisher Scientific, Waltham, MA, USA). Gels were stained with Coomassie Brilliant Blue (GenScript, Piscataway, NJ, USA). Protein concentrations were determined using the Nanodrop ND-1000 spectrophotometer adjusted by the molar extinction coefficient.

### 4.6. Western Blot

*Aedes aegypti* mosquito midguts and ovaries, as well as recombinant proteins, were separated by SDS-PAGE gel electrophoresis for Western blots. *A. aegypti* that had been fed defibrinated sheep blood (Denver Serum Company, Denver, CO, USA) were collected 48 h post-bloodmeal, and their midguts (cleaned of blood in 1× PBS) and ovaries were dissected and flash frozen on dry ice. 15 midguts or ovaries/tube were resuspended in 100 μL cold hypotonic lysis buffer (10 mM Hepes pH 7.9, 1.5 mM MgCl_2_, 10 mM KCl, 0.2 mM PMSF) and left on ice for 15 min. Samples were vortexed vigorously for 30 s and then pelleted at 1000× *g* for 15 min. The supernatant was collected as the cytoplasmic fraction. The remaining pellets were then resuspended in 100 μL solubilization buffer (15 mM Tris, 150 mM NaCl, 5 mM EDTA, 0.5% Triton X-100, 10% glycerol, 0.2 mM PMSF) and spun down at 100,000× *g*. The supernatant was collected as the nuclear fraction.

30 μg of protein was processed for SDS/PAGE separation, as described previously, and run alongside 10 µM Piwi4 PAZ or Piwi4 FL inclusion bodies. Proteins were transferred to a PVDF membrane (iBlot, Invitrogen, Waltham, MA, USA) that was blocked for 2 h at RT in blocking buffer (5% powdered milk (Carnation), 50 mM Tris-HCl pH 7.4, 150 mM NaCl, 1% Tween 20 (TBST)). Membranes were incubated O/N at 4 °C with anti-Piwi4 PAZ mouse serum (1:500 in blocking buffer), a 6xHis-tag monoclonal antibody (ThermoFisher Scientific, Waltham, MA, USA, diluted 1:5000 in blocking buffer), or anti-Histone H3 as a nuclear marker (Novus Biologicals, Littleton, CO, USA; generated in rabbit, diluted 1:1000 in blocking buffer).

Membranes were washed with TBST (3× for 10 min) and with TBS (1× 10 min) and incubated at RT for 1–2 h with goat anti-mouse or anti-rabbit antibodies conjugated to alkaline phosphatase (1 mg/mL, diluted 1:10,000). Membranes were again washed with TBST and TBS, and proteins were detected for 5–10 min using Western Blue Stabilized alkaline phosphatase substrate (Promega, Madison, WI, USA).

### 4.7. Mosquito Rearing

*A. aegypti* mosquitoes (Liverpool (LVP) strain) were reared in standard insectary conditions at the Laboratory of Malaria and Vector Research, NIAID, NIH (28 °C, 60–70% humidity, 14:10 h light/dark cycle) under the expert supervision of Karina Sewell, Andre Laughinghouse, Kevin Lee, and Yonas Gebremicale. Mosquitoes had a solution of 10% sucrose ad libitum and were offered defibrinated sheep blood (Denver Serum Company, Denver, CO, USA) in an artificial feeding system. Larva were fed Tetramin.

### 4.8. Sequence Alignment

Nucleotide and amino acid sequences were retrieved from the NCBI databases. Multiple alignments and phylogenetic trees were obtained by Clustal Omega [61] and visualized on Jalview [62].

### 4.9. Site Directed Mutagenesis

AePiwi4 PAZ protein mutants were generated using the QuikChange II Site-Directed mutagenesis kit (Agilent, Santa, Clara, CA, USA) following the manufacturer’s instructions. Primers were designed using PrimerX (https://www.bioinformatics.org/primerx/cgi-bin/DNA_1.cgi, (accessed on 14 March 2021)) and are displayed in Supplemental Table S3. The pET-17b vector containing *AePiwi4* FL was used as template for the reactions, and XL1-Blue supercompetent cells (Agilent, Santa, Clara, CA, USA) were transformed with the mutant plasmids. Mutation nucleotide sequences were confirmed by Sanger sequencing, and protein mutant sequences were confirmed by mass spectrometry.

The pET-17b vectors containing the AePiwi4 PAZ mutations were transformed into BL21(DE3) pLysS chemically competent *E. coli* cells (Thermo Fisher Scientific, Waltham, MA, USA), and all proteins were expressed and purified, as described previously.

### 4.10. Surface Plasmon Resonance (SPR)

All SPR experiments were carried out in a T100 instrument (GE Healthcare, Chicago, IL, USA) following the manufacturer’s instructions. Sensor CM5, amine coupling reagents, and HBS-P buffers were also purchased from GE Healthcare (Chicago, IL, USA). HBS-P was supplemented with EDTA (HBS-PE, 10 mM Hepes pH 7.4, 150 mM NaCl, 3 mM EDTA, and 0.005% (*v*/*v*) P20 surfactant) and was used as the running buffer while Conditioning Solution 2 (50 mM NaOH, 1 M NaCl) was used as the regeneration and conditioning solution for all experiments. Briefly, the CM5 sensor was coated 40 µg/mL neutravidin and pre-conditioned with 3 × 60 s injections of Conditioning Solution 2. ~500–1000 RUs of biotinylated RNAs were then captured to flow cells 2 or 4, which were then conditioned with 3 × 60 s injections of Conditioning Solution 2. Protein analyte was introduced unto the surface with 180 s injections (30 µL/s). Results were analyzed using the Biacore T200 Evaluation software v2.0.3 provided by GE Healthcare (Chicago, IL, USA). Equilibrium dissociation constants were calculated from steady-state binding levels (R_eq_) against molar concentration of the analyte (C). The fitted equation was R_eq_ = ((CR_max_)/(K_D_ + C)) + offset, where R_max_ = analyte-binding capacity of the surface in response units (RU) and offset = response at zero analyte concentration, which accounts for buffer-mediated effects on the refractive index. SPR experiments were carried out 2–4×.

### 4.11. Circular Dichroism

0.1 mg/mL of purified AePiwi4 PAZ WT, T39A, Y40A, T41A, or T41R in 20 mM Tris 75 mM, NaCl pH 7.4 were used for CD analyses. Continuous measurements with a pitch of 0.2 nm were recorded from 200–260 nm wavelengths with a bandwidth of 1 nm. Mean residue ellipticity was calculated with the following equation: (molecular weight of each protein in daltons/((number of amino acids − 1) × θ_λ_))/(10 × pathlength in cm × protein concentration in g/mL). All readings were normalized by subtracting with blank (buffer) mean residue ellipticity. Data were analyzed using CAPITO [51].

### 4.12. RNA Synthesis

The 3′ 2′ O-CH_3_ and 3′ 2′ OH 28 nt RNAs were synthesized by Eurofins Genomics (Louisville, KY, USA). Sequences are listed in Supplemental Table S4. RNA was resuspended at 1–2 mM in DEPC-treated water and stored at −80 °C.

### 4.13. Mouse Polyclonal Antibody Production

Polyclonal antibodies against *A. aegypti* Piwi4 PAZ were raised in mice. Mice (Balb/c; Charles River, Frederick, MD, USA) were IM immunized with 10 µg of AePiwi4 PAZ in combination with Magic Mouse Adjuvant (CD Creative Diagnostics, Shirley, NY, USA). Negative control mice were immunized with Magic Mouse Adjuvant alone. At 21 d post-immunization, mice received a 2nd booster immunization with 10 µg of AePiwi4 PAZ in combination with Magic Mouse Adjuvant (or adjuvant alone for negative control group). Blood was collected 35 d post-immunization. The antibody levels were confirmed by ELISA.

### 4.14. Mass Spectrometry

Mosquito tissue samples and recombinant proteins were prepared and separated by an SDS-PAGE gel as previously described, which was then stained with Coomassie blue. Bands of interest were excised from the gel and submitted for liquid chromatography coupled with mass spectrometry at the Research and Technology Branch (NIAID, NIH, Rockville, MD, USA). Briefly, the gel slices from the SDS-PAGE gel were cut into small pieces and subjected to in-gel trypsin digestion. The gel slices were destained to remove Coomassie blue staining and were then reduced and alkylated. After dehydration with acetonitrile and air-drying, a sequencing grade trypsin (Promega, Madison, WI, USA) solution was added onto the gel slices and was allowed to be absorbed into the gel slice. The gel slices were then incubated overnight at 30 °C for in-gel digestion. The peptides released from in-gel digestion were extracted by acetonitrile and then applied for LC-MS/MS analysis. Proteomic analyses were performed, as previously described [63].

### 4.15. IFA

Mosquito midguts or ovaries were dissected 48 h post-bloodmeal, flash fixed for 30 s in cold 4% paraformaldehyde (PFA) in PBS and cleaned of blood in cold PBS. The midguts or ovaries were then left shaking in 4% PFA in PBS O/N at 4 °C. The next day, midguts or ovaries were washed 3X in PBS and blocked O/N in blocking buffer (2% BSA, 0.5% Triton-X-100, PBS). The midguts or ovaries were then incubated with either serum from mice immunized with AePiwi4 PAZ or with Magic Mouse adjuvant alone (1:500, diluted in blocking buffer) O/N at 4 °C. The midguts or ovaries were washed with blocking buffer a minimum of 3× for 30 min and were then incubated with secondary goat anti-mouse antibodies conjugated to Alexa Fluor 594 (Thermo Fisher Scientific, Waltham, MA, USA; diluted 1:1000 in blocking buffer) for 1 h at RT. The midguts or ovaries were again washed with blocking buffer a minimum of 3× for 30 min, followed by incubation with 1 μg/mL DAPI (diluted 1:1000 in blocking buffer) and phalloidin conjugated to Alexa Fluor 488 (Thermo Fisher Scientific, Waltham, MA, USA; diluted 1:250 in blocking buffer) for 20 min at RT. The midguts or ovaries were washed 2× for 20 min with blocking buffer and 1× with 0.5% Triton-X-100 in PBS and were then mounted onto slides with ProLong Gold antifade mountant with DAPI (Thermo Fisher Scientific, Waltham, MA, USA).

### 4.16. HEK293 Cell Culture and Transfection

HEK293 cells were cultured in 35 mm dishes with a No. 15 coverslip pre-coated with Poly-D-Lysine (MatTek Life Sciences, Ashland, MA, USA). Briefly 300,000 cells were plated on individual dishes in DMEM media. The next day, the cells were transfected with transfection complex containing 500 ng of (1) SV40NLS-eGFP, (2) eGFP alone, (3) AePiwi4NLS-eGFP, or (4) AePiwi4Nterminal-eGFP in 0.5 µL Lipofectamine 3000 (Invitrogen, Waltham, MA, USA) in serum free Opti-MEM media, according to the manufacturer’s protocol. Twenty-four hours post-transfection, the cells were washed 3X with PBS and fixed with 4% PFA in PBS for 30 min at RT. The cells were then washed 3X with PBS and permeabilized with 0.5% Triton-X-100 in PBS for 30 min at RT. The cells were then stained with DAPI (1 µg/mL in 2% BSA, 0.5% Triton-X-100, PBS). The cells were visualized using a Leica Confocal SP8 microscope. Images were processed with Imaris software version 9.2.1 and post-processing was carried out in Fiji ImageJ version 1.52n for representative purposes.

### 4.17. Statistics

Surface Plasmon Resonance equilibrium curves were fitted with a non-linear regression generated by the Biacore Evaluation software v2.0.3 provided by GE Healthcare (described in “Surface Plasmon Resonance” Methods section), which were then visualized with GraphPad Prism. The equilibrium dissociation constants, calculated based on steady state, were generated by that same software. Differences between dissociation constants were compared using an unpaired two-tailed t-test with GraphPad Prism.

Quantifications of eGFP fluorescent intensities were calculated by subtracting eGFP pixel total intensity sums by average nuclear intensity sums, normalized by the number of cells, in three independent views across a slide. Nuclear surfaces were determined by DAPI display and eGFP pixel intensity values were extracted using Imaris software version 9.2.1. Differences between eGFP intensities outside of nuclear surfaces were compared using an unpaired two-tailed T-test with GraphPad Prism.

## Figures and Tables

**Table 1:** Summary of disassociation constants for AePiwi4 PAZ mutants binding 3′ 2′ O-methylated (met) or non-methylated (nmet) piRNA. Equilibrium K_D_ was calculated from steady-state binding levels R_eq_ = (CR_max_)/(K_D_ + C) + offset, where C = concentration, R_max_ = analyte-binding capacity of the surface in response units (RU), K_D_ = dissociation constant, and offset = response at zero analyte concentration. * = *p* ≤ 0.05 by unpaired t-test with WT as comparison group.

Immobilized Ligand	Binding AePiwi4 PAZ	K_D_ Values (μM)	R_max_ (RU)
3′ 2′met piRNA	WT	1.7 ± 0.7	1364 ± 3
	T39A	0.98 ± 0.2	1343 ± 2
	Y40A	5.5 ± 0.5 *	1103 ± 62
	T41A	1.4 ± 0.1	1272 ± 14
	T41R	0.57 ± 0.1	1388 ± 18
	F55A	No binding	
3′ 2′nmet piRNA	WT	5 ± 2.2	585 ± 3
	T39A	2.8 ± 0.4 *	596 ± 5
	Y40A	No binding	
	T41A	5.6 ± 1.2	580 ± 8
	T41R	2.0 ± 0.5 *	590 ± 3
	F55A	No binding	

## Supplementary Materials

The following are available online at https://www.mdpi.com/article/10.3390/ijms222312733/s1.
