# Supplementary material for: Aedes aegypti Piwi4 Structural Features Are Necessary for RNA Binding and Nuclear Localization
Source: Int J Mol Sci. 2021 Nov 25;22(23):12733. doi: 10.3390/ijms222312733 (PMC8657434; doi:10.3390/ijms222312733)
Supplement: Supplementary file 1 [file ijms-22-12733-s001.zip › Williams-IJMS-Supplemental Figs changes.pdf]

Supplemental Tables

Parameter	Value
Molecular weight (g/mol)	12,945
Net charge at neutral pH 7	5.08
pI	9.17
Average hydropathy (GRAVY)	-0.66
Aliphatic index	69
Abs280	0.82
Molar Extinction Coefficient ($M^{-1}cm^{-1}$)	10,430

Supplemental Table S1. Biophysical properties of predicted AePiwi4 PAZ. Biophysical parameters and associated values for AePiwi4 PAZ. pI = isoelectric point. Average hydropathy = the Kyte-Doolittle hydropathy value. Abs280 = the absorbance at 280 nm in an oxidized environment. Molar Extinction Coefficient = the amount of light the protein absorbs, used to calculate protein concentration.

		**Ligand K_D binding affinity (µM)**		
**Organism**	**Piwi PAZ binding partner**	**3' 2' O-met**	**3' 2' OH**	**Reference**
Drosophila	Piwi	4	unreported	Yamaguchi et al., 2020
Human	Hiwi1	6.5	16	Tian et al., 2011
Human	Hiwi2	2	12	Tian et al., 2011
Human	Hili	10	34	Tian et al., 2011

Supplemental Table S2. Summary of known Piwi PAZ binding affinities for 3' 2' O-methylated (3' 2' O-met) or non-methylated (3' 2' OH) piRNAs.

**primer name**	**sequence**	**purpose**
piwi4Nde_F	CATATGTCTGACCGTTACTCTCAAGGG	Piwi4 restriction enzyme-mediated cloning pET-17b
piwi4Xho_R	CTCGAGTCAGTGGTGGTGGT	Piwi4 restriction enzyme-mediated cloning pET-17b
piwi4_int_R	TGGTAGACATCTCCAGTACGTTCG	Piwi4 sequencing primer

piwi4pazNDE_F	CATATGCAGACATGCTACGACATCTTG	Piwi4 PAZ restriction enzyme-mediated cloning pET-17b
piwi4pazxhohis_R	CTCGAGTCAATGGTGATGGTGATGATGCGTCATCTG	Piwi4 PAZ restriction enzyme-mediated cloning pET-17b
QC-PAZ17-T41A-F	CAACAATAAAACCTATGCCATTCACGACGTCAC	Piwi4 PAZ T41A mutation
QC-PAZ17-T41A-R	GTGACGTCGTGAATGGCATAGGTTTTATTGTTG	Piwi4 PAZ T41A mutation
QC-PAZ17-T41R-F	GTTACAACAATAAAACCTATCGCATTCACGACGTCACGTTTG	Piwi4 PAZ T41R mutation
QC-PAZ17-T41R-R	CAAACGTGACGTCGTGAATGCGATAGGTTTTATTGTTGTAAC	Piwi4 PAZ T41R mutation
QC-PAZ17-Y40A-F	GTTACAACAATAAAACCGCTACCATTCACGACGTCAC	Piwi4 PAZ Y40A mutation
QC-PAZ17-Y40A-R	GTGACGTCGTGAATGGTAGCGGTTTTATTGTTGTAAC	Piwi4 PAZ Y40A mutation
QC-PAZ17-T39A-F	CGGTTACAACAATAAAGCCTATACCATTCACGAC	Piwi4 PAZ T39A mutation
QC-PAZ17-T39A-R	GTCGTGAATGGTATAGGCTTTATTGTTGTAACCG	Piwi4 PAZ T39A mutation
QC-PAZ17-F55A-F	GAAACCACTCCGGAGAGTACGGCCGATACCAAGGCCGGTAAAAC	Piwi4 PAZ F55A mutation
QC-PAZ17-F55A-R	GTTTTACCGGCCTTGGTATCGGCCGTACTCTCCGGAGTGGTTTC	Piwi4 PAZ F55A mutation
Inf-NLS-NcoI-F	GGCTGCCGCCACCATGGGATCGAGGGAACCGAGAGAACAC	Piwi4 NLS infusion-mediated cloning
Inf-NLS-NcoI-R	CCCTTGCTCACCATGGCTCCAACGCCACGGCGACTGCG	Piwi4 NLS/N terminal infusion-mediated cloning
InfNLSlong-NcoI-F	GGCTGCCGCCACCATGGGATCTGACCGTTACTCTCAAGGG	Piwi4 N terminal infusion-mediated cloning

Supplemental Table S3. Primers used in this study. Primer names (left), sequences (middle) and reason for use (right).

RNA	sequence
pclvpirnabio	[BioON]CGA[U]AAG[U]GA[U]C[U][U][U]CAGCAC[U]GCAGAA
pclvpirnametbio	[BioON]CGA[U]AAG[U]GA[U]C[U][U][U]CAGCAC[U]GCAGA[2OMe A]
scrampirnabio	[BioON] A [U] GAG GAC A [U] AAG CC [U] ACG [U] AG [U] A [U] [U] CC

Supplemental Table S4. RNA used in this study. RNA sequences used for SPR experiments. BioON = biotinylated. 2OMe = 3' 2' O-methylated.

Supplemental Figures

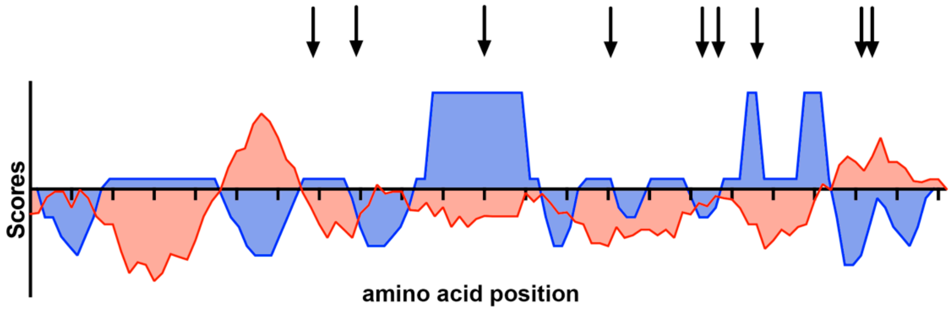

Supplemental Figure S1. Predicted AePiwi4 PAZ flexibility, hydrophobicity, and RNA binding pockets. Flexibility scores (Karplus-Schulz prediction) in blue and hydrophobicity scores (Hopp-Woods prediction) in red. Arrows indicate predicted RNA binding sites based on other crystalized Piwi structures. Graphs superimposed to each other in relation to their threshold scores.

S2A.
>AePiwi4_PAZ_DNA
catatgCAGACATGCTACGACATCTTGCGCGATTGTCAGAAGCACGATCGTAACTACA
AGGATTCGTTCAAACGTGCCGTACTTGGTGTCGTTGTACTGACCGGTTACAACAAT
AAAACCTATACCATTCACGACGTCACGTTTGAAACCACTCCGGAGAGTACGTTCGA
TACCAAGGCCGGTAAAACGTCCTTCATTGAGTATTACAAACAGAAGTACAACATTC
GCATTCGTGATCCACATCAGCCTATGTTGCTGTCGCGAGCCAAGAAACGCGATCT
GCGCGCTGGAGGCAGCGAACTCATGGCCCTTGTTCCAGAACTGTGCCAGATGAC
GCATCATCACCATCACCATTGActcgag

S2B.
>AePiwi4_PAZ_protein
MQTCYDILRDCQKHDRNYKDSFKRAVLGVVVLTGYNNKTYTIHDVTFETTPESTFDTK
AGKTSFIEYYKQKYNIRIRDPHQPMLLSRAKKRDLRAGGSELMALVPELCQMTHHHHH
H

S2C.

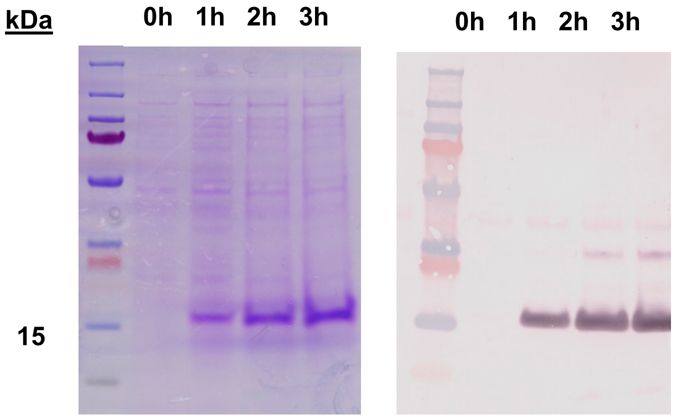

S2D.

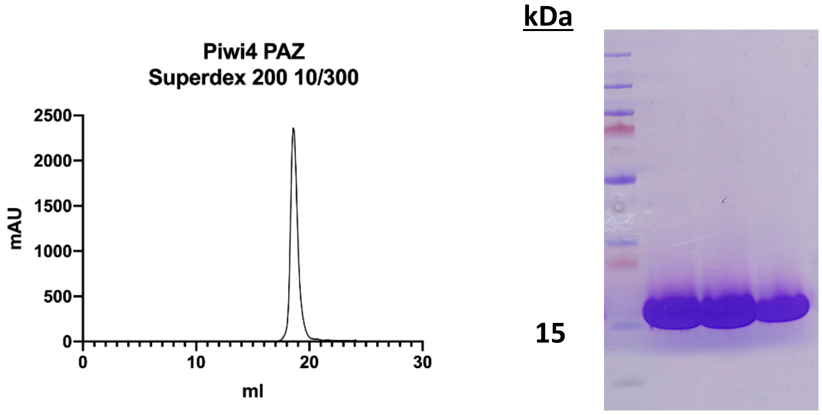

Supplemental Figure S2. AePiwi4 PAZ expression and purification. A. *AePiwi4 PAZ* nucleotide sequence, including restriction enzyme sites (lower case letters) used for cloning and a 6xHis-tag tag (underlined). **B.** AePiwi4 PAZ amino acid sequence, including 6xHis-tag. **C.** AePiwi4 PAZ expression trial in *E. coli* BL21(DE3) pLYsS cells assessed by Coomassie-stained (left) and anti-6xHis-tag western blot. Lanes indicate hours post-induction. **D.** AePiwi4 PAZ protein purification by size exclusion chromatography (left) where peak elutions were confirmed by Coomassie-stained SDS/PAGE gel (right). Expected AePiwi4 PAZ protein size = 14 kDa.

S3A.

>AePiwi4_PAZ_T39A_DNA

catatgCAGACATGCTACGACATCTTGCGCGATTGTCAGAAGCACGATCGTAACTACA
AGGATTCGTTCAAACGTGCCGTACTTGGTGTCGTTGTACTGACCGGTTACAACAAT
AAA**G**CCTATACCATTCACGACGTCACGTTTGAAACCACTCCGGAGAGTACGTTCGA
TACCAAGGCCGGTAAAACGTCCTTCATTGAGTATTACAAACAGAAGTACAACATTC
GCATTCGTGATCCACATCAGCCTATGTTGCTGTCGCGAGCCAAGAAACGCGATCT
GCGCGCTGGAGGCAGCGAACTCATGGCCCTTGTTCCAGAACTGTGCCAGATGAC
GCATCATCACCATCACCATTGActcgag

S3B.

>AePiwi4_PAZ_T39A_protein

MQTCYDILRDCQKHDRNYKDSFKRAVLGVVVLTGYNNK**A**YTIHDVTFETTPESTFDTK
AGKTSFIEYYKQKYNIRIRDPHQPMLLSRAKKRDLRAGGSELMALVPELCQMTHHHHH
H

S3C.
>AePiwi4_PAZ_Y40A_DNA
catatgCAGACATGCTACGACATCTTGCGCGATTGTCAGAAGCACGATCGTAACTACA
AGGATTCGTTCAAACGTGCCGTACTTGGTGTCGTTGTACTGACCGGTTACAACAAT
AAAACC**GC**TACCATTCACGACGTCACGTTTGAAACCACTCCGGAGAGTACGTTCGA
TACCAAGGCCGGTAAAACGTCCTTCATTGAGTATTACAAACAGAAGTACAACATTC
GCATTCGTGATCCACATCAGCCTATGTTGCTGTCGCGAGCCAAGAAACGCGATCT
GCGCGCTGGAGGCAGCGAACTCATGGCCCTTGTTCCAGAACTGTGCCAGATGAC
GCATCATCACCATCACCATTGActcgag

S3D.
>AePiwi4_PAZ_Y40A_protein
MQTCYDILRDCQKHDRNYKDSFKRAVLGVVVLTGYNNKT**A**TIHDVTFETTPESTFDTK
AGKTSFIEYYKQKYNIRIRDPHQPMLLSRAKKRDLRAGGSELMALVPELCQMTHHHHH
H

S3E.
>AePiwi4_PAZ_T41A_DNA
catatgCAGACATGCTACGACATCTTGCGCGATTGTCAGAAGCACGATCGTAACTACA
AGGATTCGTTCAAACGTGCCGTACTTGGTGTCGTTGTACTGACCGGTTACAACAAT
AAAACCTAT**G**CCATTCACGACGTCACGTTTGAAACCACTCCGGAGAGTACGTTCGA
TACCAAGGCCGGTAAAACGTCCTTCATTGAGTATTACAAACAGAAGTACAACATTC
GCATTCGTGATCCACATCAGCCTATGTTGCTGTCGCGAGCCAAGAAACGCGATCT
GCGCGCTGGAGGCAGCGAACTCATGGCCCTTGTTCCAGAACTGTGCCAGATGAC
GCATCATCACCATCACCATTGActcgag

S3F.
>AePiwi4_PAZ_T41A_protein
MQTCYDILRDCQKHDRNYKDSFKRAVLGVVVLTGYNNKTY**A**IHDVTFETTPESTFDTK
AGKTSFIEYYKQKYNIRIRDPHQPMLLSRAKKRDLRAGGSELMALVPELCQMTHHHHH
H

S3G.
>AePiwi4_PAZ_T41R_DNA
catatgCAGACATGCTACGACATCTTGCGCGATTGTCAGAAGCACGATCGTAACTACA
AGGATTCGTTCAAACGTGCCGTACTTGGTGTCGTTGTACTGACCGGTTACAACAAT
AAAACCTAT**CG**CATTCACGACGTCACGTTTGAAACCACTCCGGAGAGTACGTTCGA
TACCAAGGCCGGTAAAACGTCCTTCATTGAGTATTACAAACAGAAGTACAACATTC
GCATTCGTGATCCACATCAGCCTATGTTGCTGTCGCGAGCCAAGAAACGCGATCT
GCGCGCTGGAGGCAGCGAACTCATGGCCCTTGTTCCAGAACTGTGCCAGATGAC
GCATCATCACCATCACCATTGActcgag

S3H.
>AePiwi4_PAZ_T41R_protein
MQTCYDILRDCQKHDRNYKDSFKRAVLGVVVLTGYNNKTY**R**IHDVTFETTPESTFDTK
AGKTSFIEYYKQKYNIRIRDPHQPMLLSRAKKRDLRAGGSELMALVPELCQMTHHHHH
H

S3I.
>AePiwi4_PAZ_F55A_DNA
catatgCAGACATGCTACGACATCTTGCGCGATTGTCAGAAGCACGATCGTAACTACA
AGGATTCGTTCAAACGTGCCGTACTTGGTGTCGTTGTACTGACCGGTTACAACAAT
AAAACCTATACCATTCACGACGTCACGTTTGAAACCACTCCGGAGAGTACG**GC**CGA
TACCAAGGCCGGTAAAACGTCCTTCATTGAGTATTACAAACAGAAGTACAACATTC
GCATTCGTGATCCACATCAGCCTATGTTGCTGTCGCGAGCCAAGAAACGCGATCT
GCGCGCTGGAGGCAGCGAACTCATGGCCCTTGTTCCAGAACTGTGCCAGATGAC
GCATCATCACCATCACCATTGActcgag

S3J.
>AePiwi4_PAZ_F55A_protein
MQTCYDILRDCQKHDRNYKDSFKRAVLGVVVLTGYNNKTYTIHDVTFETTPEST**A**DTK
AGKTSFIEYYKQKYNIRIRDPHQPMLLSRAKKRDLRAGGSELMALVPELCQMTHHHHH
H

Supplemental Figure S3. AePiwi4 PAZ mutant nucleotide and protein sequences. Mutant *AePiwi4 PAZ* (**A**) T39A, (**C**) Y40A, (**E**) T41A, (**G**) T41R, (**I**) F55A nucleotide sequences, including restriction enzyme sites (lower case letters) used for cloning and a 6xhis tag (underlined). Mutant site is bolded and underlined. Mutant AePiwi4 PAZ (**B**) T39A, (**D**) Y40A, (**F**) T41A, (**H**) T41R, (**J**) F55A amino acid sequences, including 6xHis-tag. Mutation site is bolded and underlined.

S4.

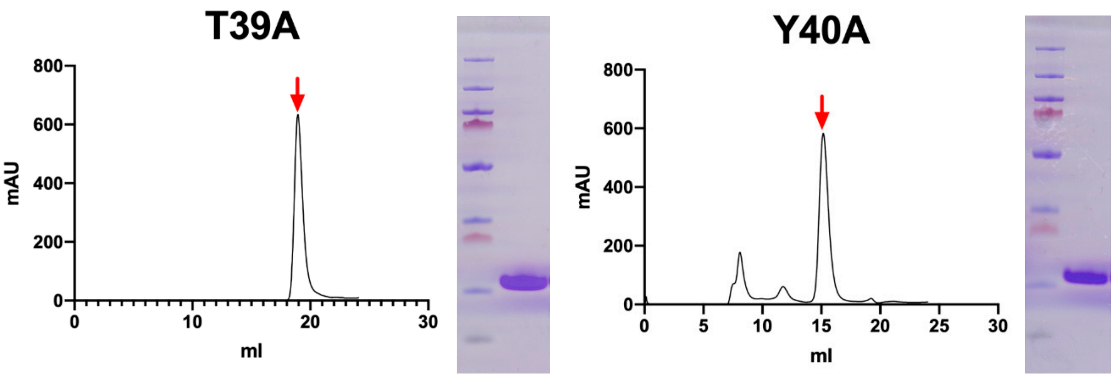

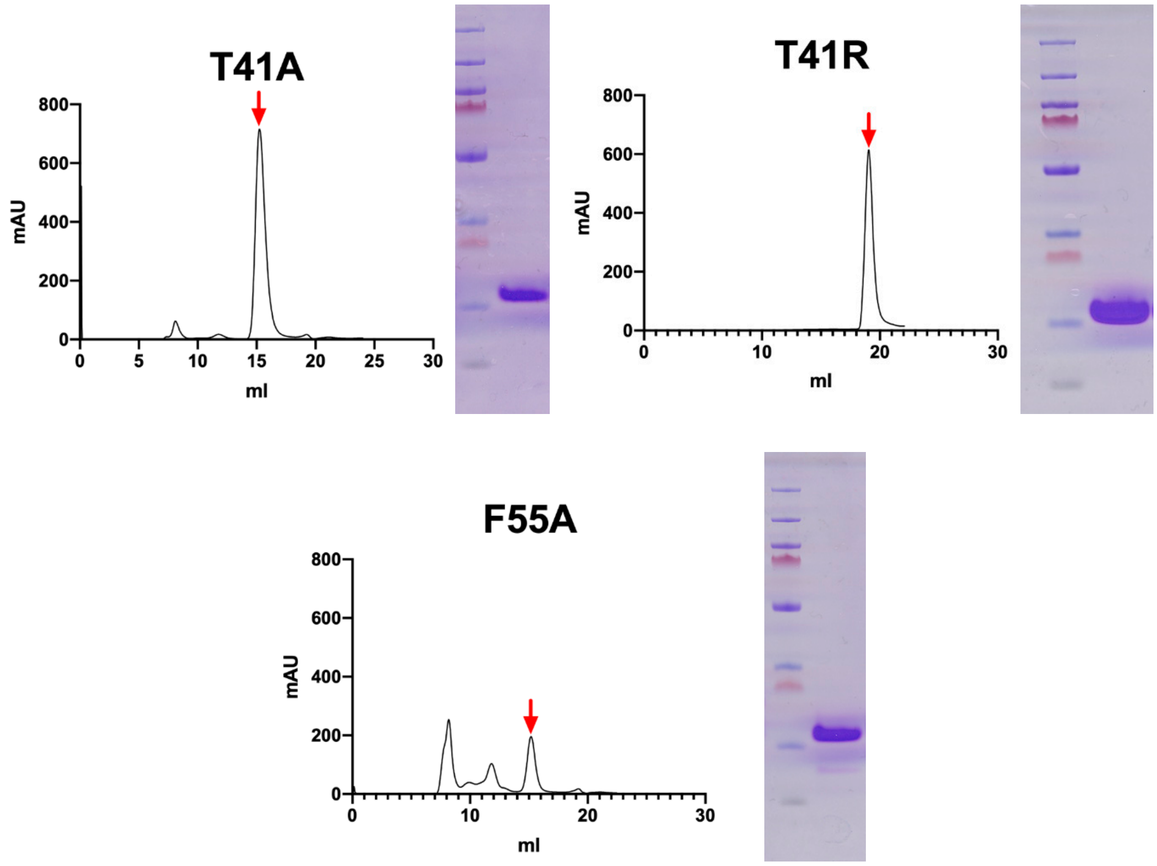

Supplemental Figure S4. Expression and Purification of AePiwi4 PAZ proteins displaying respective mutations in predicted RNA binding pockets. Size Exclusion Chromatograms and corresponding Coomassie-stained SDS/PAGE gels of purified T39A, Y40A, T41A, T41R, or F55A AePiwi4 PAZ mutants. Predicted size of all proteins are ~ 14 kDa. Red arrows indicate protein peaks of interest.

S5.

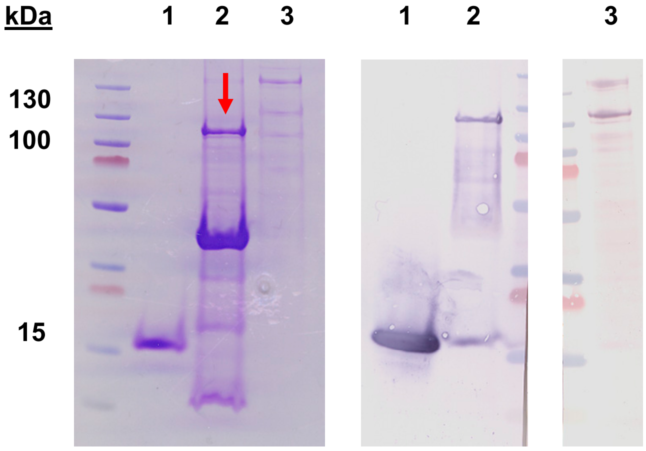

Supplemental Figure S5. Anti-AePiwi4 PAZ reacts to AePiwi4 by western blot with recombinant proteins and whole mosquito lysate. Left. Coomassie-stained SDS/PAGE gel with recombinant AePiwi4 PAZ (lane 1), recombinant AePiwi4 full length (lane 2, indicated by red arrow), and whole mosquito lysate (lane 3). **Right.** Corresponding western blot of recombinant AePiwi4 PAZ (lane 1), recombinant AePiwi4 full length (lane 2), and whole mosquito lysate (lane 3) reacts against mouse serum after immunization with recombinant AePiwi4 PAZ.

S6A.

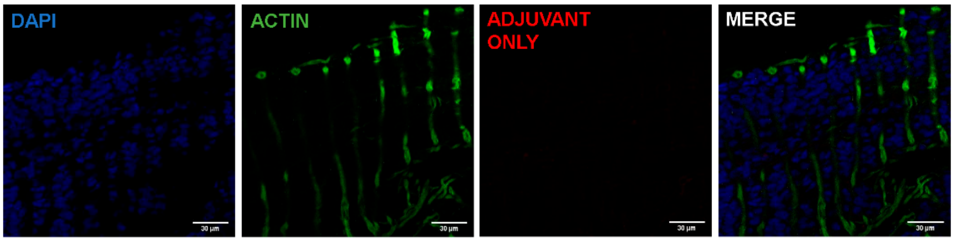

S6B.

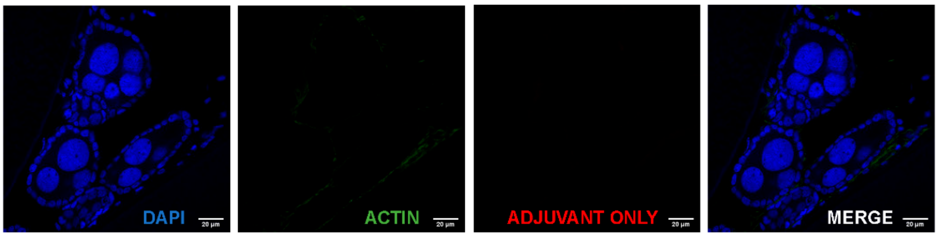

Supplemental Figure S6. Negative control IFA images of *Ae. aegypti* (A) midguts and (B) ovaries containing unfertilized eggs. IFA images of (**A**) *Ae. aegypti* midguts or (**B**) ovaries stained with mouse serum immunized with Magic Mouse adjuvant alone (red), phalloidin (green), and DAPI (blue) as negative controls for IFA analyses. Scale bars = 30 µM or 20 µM for midgut and ovary images, respectively.

S7.

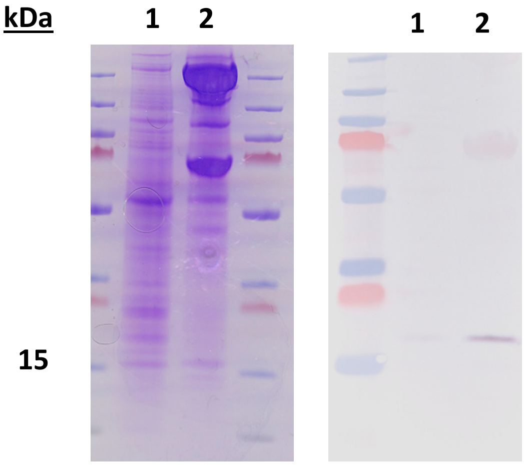

Supplemental Figure S7. Nuclear marker against *Ae. aegypti* ovary tissue cytoplasmic and nuclear fractions. Left. Coomassie-stained SDS/PAGE gel of ovary cytoplasmic (lane 1) or nuclear (lane 2) fractions display different banding patterns. **Right.** anti-H3 histone reacts against the nuclear fraction (lane 2) and not the cytoplasmic fraction (lane 1). Expected H3 size = 17 kDa.

S8A.

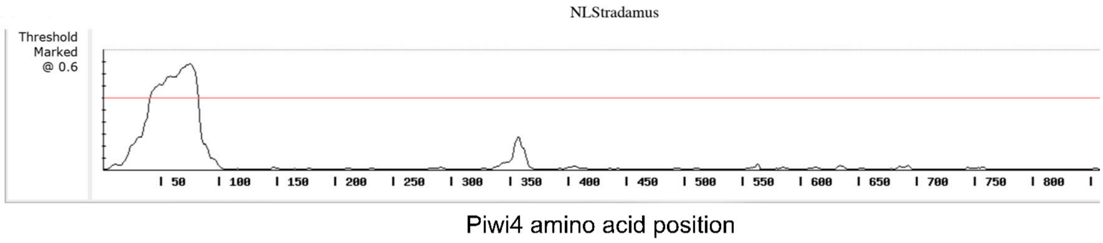

S8B.

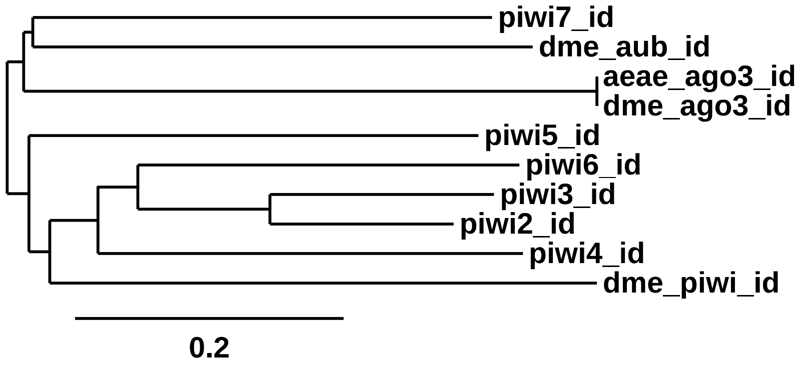

Supplemental Figure S8. Putative N-terminal NLS present in AePiwi4. A. Putative AePiwi4 NLS relative to a threshold predicted by NLStradamus (bottom) (Nguyen Ba *et al.*, 2009). **B.** Phylogenetic tree comparing the intrinsically disordered (id) domains of *Drosophila melanogaster* (dme) and *Aedes aegypti* (aeae) Piwi proteins. Scale bar indicates number of substitutions per site.
